# Mechanism matters: mortality and endothelial cell damage marker differences between blunt and penetrating traumatic injuries across three prehospital clinical trials

**DOI:** 10.1038/s41598-024-53398-1

**Published:** 2024-02-02

**Authors:** Jack K. Donohue, Danielle S. Gruen, Nidhi Iyanna, John M. Lorence, Joshua B. Brown, Francis X. Guyette, Brian J. Daley, Brian J. Eastridge, Richard S. Miller, Raminder Nirula, Brian G. Harbrecht, Jeffrey A. Claridge, Herb A. Phelan, Gary A. Vercruysse, Terence O’Keeffe, Bellal Joseph, Matthew D. Neal, Timothy R. Billiar, Jason L. Sperry

**Affiliations:** 1 Division of Trauma and General Surgery, Department of Surgery, University of Pittsburgh Medical Center, University of Pittsburgh, Pittsburgh, PA USA; 2 Department of Emergency Medicine, University of Pittsburgh, Pittsburgh, PA USA; 3 Department of Surgery, University of Tennessee Health Science Center, Knoxville, TN USA; 4 Department of Surgery, University of Texas Health San Antonio, San Antonio, TX USA; 5 Department of Surgery, JPS Health Network, Fort Worth, TX USA; 6 Department of Surgery, University of Utah, Salt Lake City, UT USA; 7 Department of Surgery, University of Louisville, Louisville, KY USA; 8 Department of Surgery, Metro Health Medical Center, Case Western Reserve University, Cleveland, OH USA; 9 Department of Surgery, University of Texas Southwestern, Dallas, TX USA; 10 Department of Surgery, University of Arizona, Tucson, AZ USA

**Keywords:** Prognostic markers, Outcomes research, Clinical trial design, Randomized controlled trials

## Abstract

Injury mechanism is an important consideration when conducting clinical trials in trauma. Mechanisms of injury may be associated with differences in mortality risk and immune response to injury, impacting the potential success of the trial. We sought to characterize clinical and endothelial cell damage marker differences across blunt and penetrating injured patients enrolled in three large, prehospital randomized trials which focused on hemorrhagic shock. In this secondary analysis, patients with systolic blood pressure < 70 or systolic blood pressure < 90 and heart rate > 108 were included. In addition, patients with both blunt and penetrating injuries were excluded. The primary outcome was 30-day mortality. Mortality was characterized using Kaplan–Meier and Cox proportional-hazards models. Generalized linear models were used to compare biomarkers. Chi squared tests and Wilcoxon rank-sum were used to compare secondary outcomes. We characterized data of 696 enrolled patients that met all secondary analysis inclusion criteria. Blunt injured patients had significantly greater 24-h (18.6% vs. 10.7%, log rank *p* = 0.048) and 30-day mortality rates (29.7% vs. 14.0%, log rank *p* = 0.001) relative to penetrating injured patients with a different time course. After adjusting for confounders, blunt mechanism of injury was independently predictive of mortality at 30-days (HR 1.84, 95% CI 1.06–3.20, *p* = 0.029), but not 24-h (HR 1.65, 95% CI 0.86–3.18, *p* = 0.133). Elevated admission levels of endothelial cell damage markers, VEGF, syndecan-1, TM, S100A10, suPAR and HcDNA were associated with blunt mechanism of injury. Although there was no difference in multiple organ failure (MOF) rates across injury mechanism (48.4% vs. 42.98%, *p* = 0.275), blunt injured patients had higher Denver MOF score (*p* < 0.01). The significant increase in 30-day mortality and endothelial cell damage markers in blunt injury relative to penetrating injured patients highlights the importance of considering mechanism of injury within the inclusion and exclusion criteria of future clinical trials.

## Introduction

The management of severe traumatic injury has undergone significant evolution over the past decade, with a focus on prevention of coagulopathy, early blood transfusion and modulation of the downstream immune response which complicates traumatic injury^1–5^. Concomitantly, there has been an increasing number of randomized clinical trials characterizing early interventions to reduce the morbidity and mortality attributable to hemorrhage and severe injury^6–9^. Despite a paucity of these trials demonstrating significant primary outcome effects, survival benefits have been demonstrated in patient subgroups with specific injury characteristics.

Patients who suffer traumatic injury represent a heterogenous population who vary widely in mechanisms of injury, demographics and injury severity^6,7,10–12^. Blunt and penetrating mechanisms of injury contribute to this heterogeneity. Several studies have demonstrated that mechanism of injury may influence the compensatory response, impact the benefit of resuscitation and generate an effect modification on risk factors for mortality following traumatic injury^13–23^. However, less is known regarding the respective morbidity, mortality time course and resultant immune response which follows blunt and penetrating injury. Despite this previous evidence, injury mechanism has not been a consistent element within the inclusion criteria of prehospital hemorrhagic shock trials^4,6–9,12,23–25^.

We sought to characterize the differences in morbidity, mortality and markers of endothelial cell damage across blunt and penetrating mechanisms of injury using harmonized data obtained from three recent prehospital, randomized, clinical trials that enrolled patients at risk of hemorrhage and severe injury. We hypothesized that there would be significant differences in the attributable morbidity, timing of mortality and the resultant endothelial cell injury across blunt and penetrating injury that may have relevance for future trial design and planning.

## Methods

### Trial designs and study populations

We performed a secondary analysis of three randomized prehospital clinical trials focused on patients at risk of hemorrhagic shock: the Prehospital Air Medical Plasma trial (PAMPer)^6^, the Study of Tranexamic Acid During Air Medical and Ground Prehospital Transport trial (STAAMP)^7^ and the Pragmatic Prehospital Type O Whole Blood Early Resuscitation trial (PPOWER)^8^.

PAMPer (NCT01818427) was a multicenter trial designed to test the effect of administering plasma to severely injured trauma patients on air ambulances before arrival to definitive trauma care. Inclusion criteria were met if patients had at least one episode of hypotension (systolic blood pressure < 90 mm Hg) and tachycardia (heart rate > 108 beats per minute) or if they had any severe hypotension (systolic blood pressure < 70 mm Hg), either before the arrival of air medical transport or any time before arrival at the trauma center. Patients were cluster-randomized to receive either standard care fluid resuscitation (crystalloid or crystalloid and packed red blood cells) or 2 units of thawed plasma followed by standard care fluid resuscitation.

The STAAMP trial (NCT02086500) was a multicenter trial that examined outcomes in severely injured trauma patients who received prehospital tranexamic acid (TXA) during air medical or ground transport. The study included patients from the scene or transferred from an outside emergency department to a participating trauma center within 2 h of injury with either hypotension (systolic blood pressure < 90 mm Hg) or tachycardia (heart rate > 110 beats per minute). Patients were double-blind-randomized to receive TXA (1 g bolus over 10 min en route to hospital) or placebo in the prehospital phase. Those in the treatment arm were then randomized to 1 of 3 in-hospital phase TXA dosing regimens (no additional TXA, 1 g of TXA infused over 8 h, or bolus of 1 g TXA followed by 1 g TXA infusion over 8 h).

The PPOWER trial (NCT03477006) was a single-center pilot trial designed to test the effect of administering low titer group O whole blood (LTOWB) to severely injured trauma patients on air ambulances before arrival to definitive trauma care. Inclusion criteria were identical to that of PAMPer. Patients were cluster-randomized to receive whole blood resuscitation or standard prehospital care fluid resuscitation (red cell transfusion and crystalloids).

All three trials employed exception from informed consent enrollment through the Emergency Exception from Informed Consent (EFIC) protocol, after a period of community consultation and public notification. PAMPer (STUDY20070132), STAAMP (STUDY19060072), and PPOWER (STUDY19080344) trials were all approved by the University of Pittsburgh Institutional Review Board and at all other study sites. Informed consent was obtained from all subjects enrolled in each of the trials. All study methods were carried out in accordance with relevant guidelines and regulations.

### Inclusion criteria

We harmonized these three trials after study completion to maximize the incidence of penetrating injuries for characterization. All patients from PAMPer and PPOWER were included in the secondary analysis. Patients from STAAMP were included if they met the inclusion criteria of PAMPer and PPOWER.

### Sample collection and measurement

Blood samples were collected from PAMPer and STAAMP trial patients upon hospital admission (the first blood draw, referred to as 0 h) and assayed for 7 endothelial cell damage markers. Blood samples were not collected or assayed for PPOWER trial patients.

Damage markers adiponectin, histone-complexed DNA (HcDNA) fragments, human S100 calcium-binding protein A10 (S100A10), soluble urokinase receptor (suPAR), syndecan-1, thrombomodulin (TM) and vascular endothelial growth factor (VEGF) were assayed by commercially available immunoassays in EDTA plasma according to the manufacturer’s recommendations as previously reported^26^. We analyzed soluble biomarkers representing damage to the glycocalyx (syndecan-1, catalog 950.640.192, lot no. 0138-62+0138-66, Nordic Biosite ApS), endothelium (TM, catalog 850.720.192, lot no. 0141-47, Nordic Biosite ApS) and endothelial tight-junction (VEGF-R1/Flt-1, catalog DVR100C, lot no. P186961, Bio-Techne). We also analyzed markers of cell death as cell-free DNA (HcDNA, catalog 11774425001, lot no. 29876600, Sigma-Aldrich), immunologically active endothelial cells (suPAR, catalog E001, lot no. XS2141, suPARnostic, ViroGates), mediators of fibrinolysis (S100A10, catalog abx152996, lot no. E1905813M, Abbexa Ltd.) and an adipokine related to endothelial function (adiponectin, catalog DRP300, lot no. P186579, Bio-Techne) as previously described^27^.

### Outcomes

The primary outcome for the current secondary analysis is 30-day mortality. Secondary outcomes included 24-h mortality, units of in-hospital blood components administered within 24-h, endothelial cell damage markers at hospital admission, nosocomial infection and multiple organ failure (MOF). The Denver MOF Score was used to rate the dysfunction of four organ systems (pulmonary, renal, hepatic and cardiac), which are evaluated daily throughout the patient’s ICU stay and graded on a scale from 0 to 3, with the total score ranging from 0 to 12^28^.

### Statistical analysis

Descriptive statistics characterized the demographics and injuries of the patients and outcomes of interest. A Shapiro–Wilk test was conducted on all continuous variables to test for normality. Categorical variables were presented as frequencies and percentages and tested using the Chi-squared test. Continuous variables were expressed as medians with interquartile ranges (IQRs) and were tested using Wilcoxon rank-sum.

We evaluated 24-h and 30-day mortality across blunt and penetrating mechanism of injury using Kaplan–Meier via log rank comparison. To verify these unadjusted findings, we then performed a multivariate analysis of survival with the use of a Cox proportional-hazards model, to evaluate the mechanism of injury effect (blunt vs. penetrating) with adjustment for possible confounding factors. The model was generated for the primary outcome in patients with blunt injury. Patient demographics, prehospital vital signs, prehospital interventions, injury severity score and traumatic brain injury (defined as head abbreviated injury score > 2) were assessed. In the final model, only covariates with a p-value < 0.2 were utilized to prevent over fitting of the model. An identical model was utilized for all Cox-regression analyses.

To assess endothelial cell damage marker concentrations among blunt and penetrating injured trauma patients, we built seven generalized linear models (GLM). This was necessary due to the distinct factors that may influence each of the seven endothelial cell damage markers. The aforementioned methodology for the Cox-regression analyses was used to build the seven GLMs. Biomarkers were measured at hospital admission and 24 h. We evaluated variance inflation factors to ensure that the variance of our regression coefficients was not due to multicollinearity. Statistical significance was determined at the *p* < 0.05 level (2-sided). All data was analyzed using STATA version 17.0.

### Ethical approval and consent to participate

PAMPer (STUDY20070132), STAAMP (STUDY19060072), and PPOWER (STUDY19080344) trials were all approved by the University of Pittsburgh Institutional Review Board and at all other study sites. Informed consent was obtained from all subjects enrolled in each of the trials.

## Results

In this harmonized prehospital plasma study cohort (PAMPer-n = 494, STAAMP-n = 120, PPOWER-n = 82; total n = 696), patients were severely injured with a median injury severity of 21 (IQR 11, 29), a median prehospital systolic blood pressure of 70 mmHg (IQR 62, 82 mmHg) and a median Glasgow Coma Score (GCS) of 11 (IQR 3,15).

Just over 80% of injuries for the study cohort were due to a blunt mechanism of injury (n = 575) with the remaining resulting from penetrating mechanism (n = 121). The patients who suffered from both blunt and penetrating injuries were excluded from analysis. Blunt injured patients and penetrating injured patients were evenly distributed across all three trials. Most blunt injuries were secondary to motor vehicle collisions while penetrating injuries were primarily firearm injury and stabbings (Table 1). There were also important differences in the study cohort across those who suffered blunt and penetrating mechanisms of injury. Blunt injured patients were older, had higher injury severity score overall and had lower prehospital GCS. Penetrating injured patients were more likely to be male, more racially diverse and more likely to receive prehospital blood. The two cohorts did not differ upon comparison of blood component transfusions within 24-h.

**Table 1:** Baseline characteristics for harmonized study cohort stratified by mechanism of injury.

Variable	Blunt (n = 575)	Penetrating (n = 121)	*p* value
Classification of blunt injury, n (%)			
Motor vehicle	311 (54.09)		
Motorcycle	104 (18.09)		
Pedestrian/cyclist	39 (6.79)		
Fall	65 (11.30)		
Other	56 (9.74)		
Classification of penetrating injury, n (%)			
Firearm		69 (53.02)	
Stabbing		44 (36.36)	
Other		8 (6.61)	
Full cohort, n (%)			
PAMPer	406 (70.61)	88 (72.73)	
STAAMP	100 (17.39)	20 (16.53)	
PPOWER	69 (12.00)	13 (10.74)	
Age, median (IQR)	47 (30, 62)	36 (27, 50)	< 0.001
Male, n (%)	390 (67.83)	103 (85.12)	< 0.001
Race, n (%)			
White	526 (91.48)	77 (63.64)	< 0.001
Black	22 (3.83)	36 (29.75)	
Asian	2 (0.35)	0 (0.00)	
Other	8 (1.39)	2 (1.65)	
Unknown	17 (2.96)	6 (4.96)	
Injury severity score			
Median (IQR)	22 (14, 33)	10 (6, 18)	< 0.001
ISS ≥ 16, n (%)	417 (72.52)	45 (37.19)	< 0.001
Abbreviated injury score			
Head, median (IQR)	2 (0, 3)	0 (0, 0)	< 0.001
Head, ≥ 3, n (%)	243 (42.26)	14 (11.57)	< 0.001
Face, median (IQR)	0 (0, 1)	0 (0, 0)	< 0.001
Face, ≥ 3, n (%)	32 (5.57)	3 (2.48)	0.158
Chest, median (IQR)	3 (0, 3)	0 (0, 3)	< 0.001
Chest, ≥ 3, n (%)	321 (55.83)	36 (29.75)	< 0.001
Abdomen, median (IQR)	2 (0, 3)	0 (0, 2)	< 0.001
Abdomen, ≥ 3, n (%)	155 (26.96)	24 (19.83)	0.103
Extremity, median (IQR)	2 (0, 3)	0 (0, 2)	< 0.001
Extremity, ≥ 3, n (%)	197 (34.26)	22 (18.18)	0.001
Skin, median (IQR)	1 (0, 1)	1 (0, 1)	0.395
Skin, ≥ 3, n (%)	18 (3.13)	4 (3.31)	0.920
24 h transfusions, units, median (IQR)			
Total	4 (0, 13)	4 (1, 14)	0.594
RBC	3 (0, 7)	3 (1, 7)	0.575
Plasma	0 (0, 3)	0 (0, 3)	0.969
Platelets	0 (0, 1)	0 (0, 0)	0.599
Prehospital interval			
Minutes, median (IQR)	41 (33, 52)	41 (32, 54)	0.645
≤ 20 min, n (%)	13 (2.26)	2 (1.65)	0.676
Prehospital vital signs, median (IQR)			
Heart rate	117 (102, 128)	115 (107, 126)	0.609
Systolic blood pressure	71 (63, 82)	68 (60, 80)	0.065
Glasgow coma score	11 (3, 15)	12 (4, 15)	0.020
Prehospital intervention			
Blood, n (%)	187 (32.52)	54 (44.63)	0.011
Intubation, n (%)	279 (48.52)	52 (42.98)	0.267
CPR, n (%)	33 (6.52)	6 (5.56)	0.709

Patients with blunt mechanisms of injury had significantly greater 24-h mortality (18.6% vs. 10.7%, *p* = 0.04) and 30-day mortality (29.7% vs. 14.0%, *p* < 0.01) as compared to those patients with penetrating mechanisms of injury (Table 2). Blunt injured patients also had longer ICU length of stay and mechanical ventilator days relative to penetrating injured patients. In addition, there were higher rates of nosocomial infection (NI) in patients suffering from blunt injury relative to penetrating injury. Although there was no difference in the rate of MOF across injury mechanism, blunt injured patients had significantly higher maximum multiple organ failure (MOF) score.

**Table 2:** Injury complications for harmonized study patients stratified by mechanism of injury.

Variable	Blunt (n = 575)	Penetrating (n = 121)	*p* value
24-h mortality, n (%)	107 (18.61)	13 (10.74)	0.037
30-day mortality, n (%)	171 (29.74)	17 (14.05)	< 0.001
ICU length of stay, median (IQR)	5 (1, 12)	2 (0, 5)	0.001
Mechanical ventilator days, median (IQR)	2 (1, 9)	1 (1, 4)	0.004
NI, n (%)	127 (22.09)	15 (12.40)	0.016
MOF, n (%)	278 (48.43)	52 (42.98)	0.275
Denver MOF components, median (IQR)			
Pulmonary	2 (0, 3)	1 (0, 3)	0.061
Renal	0 (0, 0)	0 (0, 0)	0.933
Hepatic	0 (0, 0)	0 (0, 0)	0.508
Cardiac	2 (0, 3)	1 (0, 3)	0.005
Denver MOF score, median (IQR)	3 (1, 5)	2 (0, 3)	< 0.001

**Figure 1:**
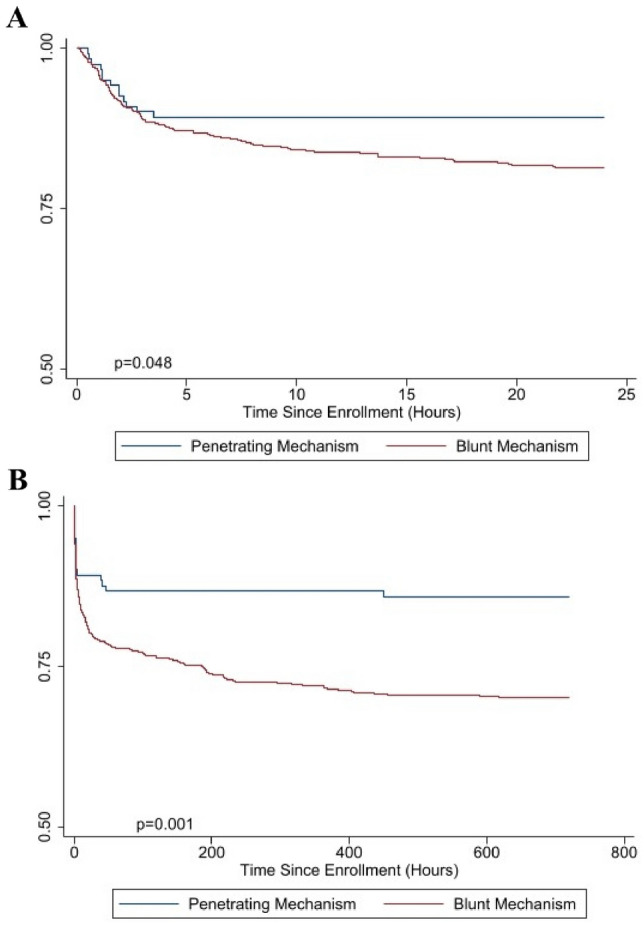
(**A**) Kaplan–Meier survival analysis comparing blunt and penetrating mechanisms of injury at 24 h. (**B**) Kaplan–Meier survival analysis comparing blunt and penetrating mechanisms of injury at 30 days.

We then performed survival analysis with Kaplan–Meier for 24-h and 30-day mortality to determine when survival differences occurred for each mechanism of injury subgroup (Fig. 1). This analysis revealed a significant separation within 24 h that persisted out to 30 days for blunt injured patients (log rank *p* = 0.048, log rank *p* = 0.001).

Multivariate analysis of survival with the use of a Cox proportional-hazards model verified that after adjusting for all clinically and statistically significant covariates that blunt mechanism of injury was independently associated with mortality at 30-days (HR 1.84, 95% CI 1.06–3.20, *p* = 0.029), but not 24-h (HR 1.65, 95% CI 0.86–3.18, *p* = 0.133) (Table 3).

**Table 3:** Multivariate Cox proportional-hazards model for 24-h and 30-day mortality.

Variable	Hazard ratio	CI 95%	*p* value
24-h mortality			
Blunt injury	1.65	0.86, 3.18	0.133
Injury severity score	1.00	0.98, 1.01	0.566
Age	1.00	0.99, 1.01	0.998
Female	1.30	0.82, 2.08	0.265
Prehospital intubation	5.18	2.52, 10.65	< 0.001
Prehospital CPR	3.85	2.26, 6.56	< 0.001
Prehospital systolic blood pressure	0.98	0.97, 0.99	< 0.001
Prehospital blood	1.30	0.87, 1.96	0.206
Prehospital glasgow coma score	0.95	0.89, 1.01	0.080
30-day mortality			
Blunt injury	1.84	1.06, 3.20	0.029
Injury severity score	1.01	1.00, 1.02	0.101
Age	1.01	1.00, 1.02	0.007
Female	1.31	0.91, 1.88	0.146
Prehospital intubation	2.83	1.73, 4.63	< 0.001
Prehospital CPR	3.14	1.98, 4.96	< 0.001
Prehospital systolic blood pressure	0.98	0.98, 0.99	< 0.001
Prehospital blood	1.33	0.96, 1.84	0.089
Prehospital glasgow coma score	0.92	0.88, 0.97	0.001

When we performed multivariate linear regression to characterize endothelial cell damage markers, elevated levels VEGF, syndecan-1, TM, S100A10, suPAR and HcDNA were independently associated with blunt mechanism of injury at the earliest sampling soon after admission (Table 4). Levels of adiponectin were not different across groups. Elevated endothelial cell damage markers patterns at 24-h after admission were no longer associated with blunt mechanism of injury.

**Table 4:** Model estimated coefficients of blunt injury relative to penetrating injury for hospital admission endothelial markers.

Variable	Coefficient	CI 95%	*p* value
Admission			
VEGF	202.83	57.87, 347.79	0.006
Syndecan-1	26.07	8.44, 43.69	0.004
TM	1.56	0.70, 2.43	< 0.001
S100A10	1.00	0.08, 1.93	0.034
SuPAR	0.68	0.25, 1.11	0.002
HcDNA	8.02	0.01, 16.03	0.050
Adiponectin	819.17	− 520.63, 2158.98	0.230
24-h			
VEGF	110.73	− 30.96, 252.42	0.125
Syndecan-1	11.61	− 6.01, 29.23	0.196
TM	0.58	− 0.73, 1.88	0.384
S100A10	0.29	− 0.41, 0.98	0.419
SuPAR	0.72	− 0.01, 1.44	0.052
HcDNA	4.07	− 0.70, 8.85	0.094
Adiponectin	818.77	− 299.36, 1936.90	0.151

## Discussion

Initiating prehospital resuscitation strategies as close to the time of injury as feasible has great potential to improve outcomes in patients at risk of hemorrhage and attributable mortality. These types of interventions are successfully being studied using high level clinical trials with variable outcome benefits^6–8,12,25^. Interventions being studied vary in their hypothesized mode of action. Tailoring the study cohort and minimizing heterogeneity may be paramount in demonstrating the efficacy and applicability of an intervention. The results from the current analysis derived from three harmonized prehospital clinical trials conducted in the United States demonstrate that blunt injury is associated with distinct clinical outcomes and endothelial injury marker trajectories relative to penetrating injury and that these differences may be important when similar clinical trials are planned in the future.

Blunt and penetrating mechanisms of injury both pose a risk of hemorrhage, but their demographics and management strategies have been shown to vary^29–33^. The incidence of blunt versus penetrating mechanisms of injury differ based upon the environment of injury (combat versus civilian setting) and the country where injury occurs^34,35^. Previous work has demonstrated that mechanism of injury may influence the compensatory response, impact the benefit of resuscitation, and generate an effect modification on risk factors for mortality following traumatic injury^13–23^. Of this body of work, several studies have demonstrated that mechanism of injury impacts the efficacy and safety of hydroxyethyl starch resuscitation, such that the use of hydroxyethyl starch is beneficial and safe in the resuscitation of penetrating injured patients, but not blunt injured patients^13,18,22^. This differing response to treatment has also been observed with controlled fluid resuscitation strategy, where an early survival advantage was seen in blunt but not penetrating injured patients^23^. Importantly, modulating factors for mortality such as sex hormones and glucose levels have also been shown to demonstrate varying effects based on mechanism of injury^15,17^. Similarly risk factors for venous thromboembolism are different between mechanisms of injury^19^. Despite these differences, mechanism of injury has not been a consistent component of inclusion criteria for clinical trials following traumatic injury^4,6–9,12,23–25^.

Blunt relative to penetrating mechanism of injury is also associated with many underlying differences that may contribute to heterogeneity of an enrolled study cohort^16^. Characteristics such as urban versus rural injury location^36^, air medical versus ground transport^37^, transfer origin^38^, prehospital transport time^11^ and socioeconomic factors^39^ are known to differ across mechanism of injury and represent inherent confounders when comparing blunt versus penetrating injury in any cohort of injured patients. Penetrating injury may also be associated with an enrollment bias. It is known that a large proportion of patients with penetrating injury are not transferred to definitive trauma care due to death at the scene^40^. These severely injured patients would not be enrolled without vital signs during transport and may be an underlying reason for lower injury severity in those that are enrolled in hemorrhagic shock trials. Understanding potential confounding factors across blunt and penetrating mechanisms of injury is critical to understand and essential for conducting future successful clinical trials post-injury.

Differences in endothelial cell damage marker patterns have not been adequately characterized across between blunt and penetrating mechanisms of injury. There were significantly higher levels of endothelial cell damage markers at admission in blunt injured patients relative to penetrating injured patients. HcDNA, S100A10 and suPAR have all been hypothesized to be associated with endothelial cell damage or function^41–43^. It is also hypothesized that adiponectin, produced by adipocytes, may play a restorative role in endothelial function.^26,44,45^ Syndecan-1, TM and VEGF have been associated with endothelial cell damage following trauma^26,27,46,47^. Because of the potential relationship between these markers and endothelial function, we categorized these seven markers as endothelial cell damage markers for the purposes of this study. The independent association of blunt injury with elevated endothelial cell damage markers has implications for future interventional clinical trials with immune associated outcomes.

### Limitations

There are limitations to this secondary analysis. Although the three studies were harmonized and derived from three prospective randomized clinical trials, there were important differences in the study cohorts and the protocols followed. Most important was the differences in prehospital transport time and mortality risk across the studies. Although we controlled for relevant differences via a robust statistical approach and harmonized inclusion criteria, the potential of residual confounding exists. The enrolled number of patients in the three clinical trials were different and the results from the current secondary analysis may be primarily driven by the trial with the largest enrolled population. Although the penetrating cohort was derived from combining three studies, drawing definitive conclusions from this smaller penetrating subgroup may still be limited. Enrolled patients were primarily transferred to definitive trauma care via air medical transport and the current associations may not be applicable to other methods of prehospital transport. Although all data were collected prospectively, the acuity of these patients upon presentation limited the collection of time sensitive data, including but not limited to laboratory tests resulting in missingness. Although the missingness did not vary across any of the groups that were compared, missing data represents a significant limitation in interpreting the endothelial cell biomarker data.

## Conclusions

In conclusion, blunt injured patients at risk of hemorrhage from the current harmonized trial data are more severely injured, have higher mortality and higher admission levels of endothelial cell damage markers relative to penetrating injured patients. Considering mechanism of injury when planning a study’s inclusion and exclusion criteria for a trauma trial may be essential and can promote alignment of the hypothesized mechanisms responsible for a treatment and enrolled population receiving benefit. These results, in the context of previous work, have important relevance to the future conduct of clinical trials investigating prehospital interventions post-injury.

## Data Availability

Following publication of the primary and all secondary analyses detailed in study protocols, individual de-identified data will be available upon request and approval of the proposed use of the data after 3 years of the close of the trial. The trial protocol, statistical analysis plan embedded in the protocol and the trial publications are available on-line. Requests should be sent to the corresponding author.
